# Genus Brassica By-Products Revalorization with Green Technologies to Fortify Innovative Foods: A Scoping Review

**DOI:** 10.3390/foods12030561

**Published:** 2023-01-27

**Authors:** Francisco Artés-Hernández, Lorena Martínez-Zamora, Marina Cano-Lamadrid, Seyedehzeinab Hashemi, Noelia Castillejo

**Affiliations:** 1 Postharvest and Refrigeration Group, Department of Agronomical Engineering and Institute of Plant Biotechnology, Universidad Politécnica de Cartagena, 30203 Cartagena, Murcia, Spain; 2 Department of Food Technology, Nutrition, and Food Science, Faculty of Veterinary Sciences, University of Murcia, 30071 Espinardo, Murcia, Spain

**Keywords:** reutilization, food loss and waste, circular economy, *Brassicaceae*, fortification, ultrasound, microwave, enzymes, extraction, reformulation

## Abstract

Food losses and waste reduction are a worldwide challenge involving governments, researchers, and food industries. Therefore, by-product revalorization and the use of key extracted biocompounds to fortify innovative foods seems an interesting challenge to afford. The aim of this review is to evaluate and elucidate the scientific evidence on the use of green technologies to extract bioactive compounds from Brassica by-products with potential application in developing new foods. Scopus was used to search for indexed studies in JCR-ISI journals, while books, reviews, and non-indexed JCR journals were excluded. Broccoli, kale, cauliflower, cabbage, mustard, and radish, among others, have been deeply reviewed. Ultrasound and microwave-assisted extraction have been mostly used, but there are relevant studies using enzymes, supercritical fluids, ultrafiltration, or pressurized liquids that report a great extraction effectiveness and efficiency. However, predictive models must be developed to optimize the extraction procedures. Extracted biocompounds can be used, free or encapsulated, to develop, reformulate, and/or fortify new foods as a good tool to enhance healthiness while preserving their quality (nutritional, functional, and sensory) and safety. In the age of recycling and energy saving, more studies must evaluate the efficiency of the processes, the cost, and the environmental impact leading to the production of new foods and the sustainable extraction of phytochemicals.

## 1. Background

Considering that the food supply chain can be divided into four main stages (primary production, processing, retail/distribution, and consumption), the Food and Agriculture Organization of the United Nations (FAO) defines ‘food loss’ as food discarded along the primary production and processing stages, while ‘food waste’ is obtained during retail/distribution and consumption [1]. During the last few decades, the European Commission has adopted a set of proposals to make the European Union’s (EU) policies fit for reducing net greenhouse gas emissions by at least 55% by 2030, compared to levels in the 1990s [2]. Around 20% of food produced in the EU is lost or wasted, accounting for 8–10% of global [3] and about 6% of total EU greenhouse gas emissions [4], a huge environmental impact. Eighty-eight million tons of food losses and waste are generated each year in the EU, with estimated costs of EUR 143 billion [4]. According to a mass flow analysis, a total of 129 Mt of food losses and waste were generated in the EU in 2011 [5], of which 79% were plant-based products. Among plant-based by-products, 30% and 28% were fruit and vegetables (F and V), respectively [5].

In the primary production steps, the main food losses are due to: (i) non-harvested edible products, (ii) edible products left in the field, (iii) edible products harvested but not sold, (iv) rotten F and V, (v) products damaged due to handling, and (vi) products stored in poor conditions, among others. During handling and processing, issues throughout the process (inefficiencies, contamination), inedible parts (peels, leaves, pomace), and food damaged by inappropriate packaging are also generated as food losses. In the distribution step, food waste appears mainly due to the lack of refrigeration, expired dates, unsold food, and food rejected after quality controls [6]. It has been reported that the higher food waste along the food supply chain of developed countries is generated in the consumption stage, with 46% of the total being 41% F and V, followed by primary production (25% of the total being 76% F and V), and processing and manufacturing (24% of the total being 20% fruit) [5,6,7].

Reducing food loss and waste has an enormous potential to minimize the resources we use to produce foods [2], being an integral part of the Farm to Fork Strategy action plan described by the EU [8]. In this sense, this plan seeks to create a food chain that is useful for consumers, producers, the climate, and the environment. It will ensure sustainable food production and guarantee food security by promoting sustainable consumption and reducing food waste, which will be achieved through research, innovation, and investment in new technologies capable of facilitating this transition [8]. Recently, there have been some strategies, such as the production of biomass and animal feed, but they do not guarantee efficient by-product use. To remain a competitive horticultural sector, especially for brassicas, it is necessary to develop appropriate postharvest strategies to increase shelf life and, on the other hand, to generate a model for the revalorization of the by-products by using ‘Green Technologies’ [8].

The selected strategies must change the sector’s production model and evolve toward a more sustainable circular economy, providing more added value and competitiveness. These strategies can focus on obtaining potential ingredients for the food and/or pharmaceutical industries. Reuse of by-products and recycling of food waste facilitate the generation of a low-carbon economy by using renewable resources, including their environmental and economic benefits, and improving the efficiency of the food industry.

Thus, several technologies have been developed to reduce costs and make possible more sustainable production processes to optimize the extraction of key bioactive compounds from by-products. Among them, due to the thermal instability of some biocompounds, it is essential to focus on innovative non-thermal ‘Green Technologies’ such as photo-treatments to increase their content, ultrasound (USAE), microwave (MWAE), enzymatic-assisted extraction (EAE), and other technologies such as supercritical fluids, ultrafiltration, and pressurized liquids.

The Brassica genus (*Brassicaceae* family) is a large group of primarily herbaceous plants, one of the most important crops after soybean in world oilseed production, and as fresh vegetables, they are widely consumed throughout the year as part of salads or after cooking. This genus includes various types of well-known species such as cabbage, broccoli, brussels sprouts, kale, kohlrabi, pak choi, rape, turnip, mustard, and cress [9]. These species provide edible roots, leaves, stems, buds, flowers, and seeds. Several authors have previously considered vegetables to be the most important category of all foods, as they form the foundation of global food supplies and are economically significant all over the world. Brassica plants are also distinguished from other vegetable plants by their high functional (phenolic and organosulfur compounds) and nutritional properties [10].

The present review aims to evaluate the published scientific evidence on the use of green technologies to increase the content and/or improve the extraction of bioactive compounds from Brassica by-products and their further application into several food matrices in a circular economy (scoping review). For this purpose, a literature review was conducted, focusing on abiotic stresses to by-products before the bioactive compound’s extraction, and USAE, MWAE, and EAE technologies to improve the extraction from Brassica by-products.

## 2. Brassica By-Products and Their Added-Value Compounds

Harvesting broccoli generates a huge number of by-products, mainly leaves, and stalks. Broccoli leaves represent 50% of total plant biomass. Furthermore, compared to broccoli florets or stalks, broccoli leaves have higher total phenolic content (TPC), antioxidant activity (TAC), chlorophylls, and vitamins (E and K), while showing similar glucosinolate (GLS) content [11,12].

In a recent study about valorization pathways, techno-economic constraints, and environmental assessment, information was included about different Brassica processing wastes, such as broccoli (leaves and stalks), cabbage waste, and cauliflower (florets and leaves) [7]. It is shown that the bioactive compounds (GLS, phenolic acids, and flavonoids) can be jointly extracted with other nutrients (vitamins, minerals, and trace elements) using conventional or green technologies. Other phytonutrients and pigments, such as carotenoids, can be extracted from other brassicas, such as cabbage waste. In addition, pectin and dietary fiber can be extracted, whose main sources are the non-edible parts of these vegetables (roots, stalks, florets, or even the pulp obtained from the processing) [11,12].

The main bioactive compounds found in Brassica are the GLS, from which isothiocyanates are biosynthesized and reported to be potent anticarcinogens and antimutagens, as sulforaphane (SFN) or sulforaphene are [13,14,15]. In fact, all the Brassicas present a predominating GLS in their composition, whose accumulation depends on the genotype, variety, cultivar (cv.), growing conditions, developmental stage, type of plant tissue, and postharvest handling. The main GLS in broccoli (*Brassica oleracea* var*. italica*) and cauliflower (*Brassica oleracea* var*. botrytis*) are glucoraphanin and glucobrassicin [16], while in kale (*Brassica oleracea* var*. sabellica*) they are sinigrin, glucoiberin, and glucobrassicin [17], or in cabbage (*Brassica oleracea* var. *capitata*) are sinigrin and glucoiberin [18,19]. Other Brassicas, such as mustard (*Sinapis alba*, *Brassica alba,* or *Brassica nigra*), are rich in glucosinalbin [20], while in radish (*Raphanus sativus*) are glucoraphanin and glucoraphasatin [21,22].

**Figure 1:**
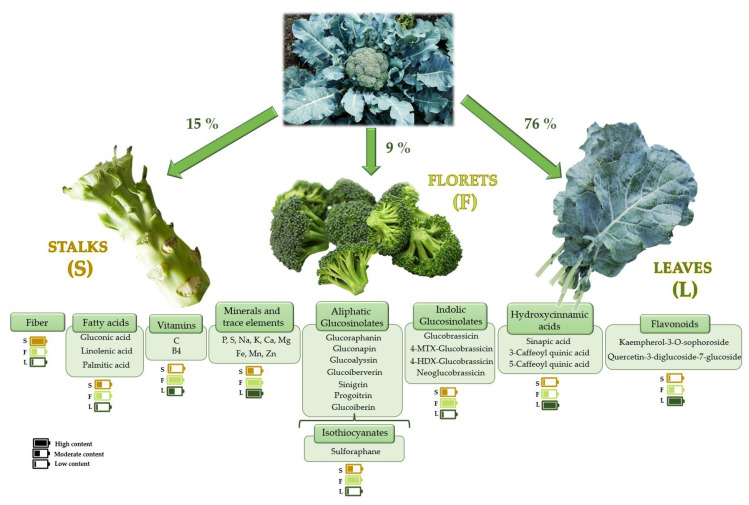
Classification of the main bioactive compounds in broccoli by-products. The content is based on published literature [24,25,26,27].

Figure 1 shows the bioactive compound classification of broccoli by-products, which are the most produced worldwide and consumed Brassicas, and hence the main source of by-products. Nevertheless, there are other Brassica by-products with different compositions, i.e., those rich in anthocyanins and carotenoids, as in the case of red cabbage [19], or flavonoids and dietary fiber from cauliflower [23].

## 3. Materials and Methods

Scopus was used for searching the documents included in the present review. The purpose of a scoping review is to provide an overview of the available evidence on a topic by compiling and evaluating the information. “Brassica”, “extraction”, and “by-product” were used as the search words, and the following items were also used: “broccoli” OR “ultraviolet light” OR “blanching” OR “cutting powdering” OR “enzymatic treatment” OR “ultrasound” OR “microwave”. The inclusion criteria were papers published in indexed JCR journals (Q1–Q4), while the exclusion criteria were books, reviews, and scientific articles published in non-indexed JCR journals. To include the most known *Brassicaceae*, all information related to Brassica extraction by-products was verified by including the terms “broccoli”, “cauliflower”, “kale”, “mustard”, “wasabi”, “kohlrabi”, “cabbage”, and “radish”. Additionally, Scopus was used to search for studies on healthy or functional foods elaborated with Brassica by-products. “Brassica” AND “by-products”, “Broccoli” AND “by-products”, “Cauliflower” AND “by-products”, “Kale” AND “by-products”, or “Mustard” AND “by-products” were used as the search words. For data curation, the title, and abstracts of the works found were analyzed and classified depending on their relevance with the help of Excel. First, the papers not focused on the studied field were excluded. Then the potential papers were subjected to an exhaustive analysis, in which all the papers were checked for inclusion criteria.

## 4. Abiotic Stresses to Enhance Bioactive Compounds in Brassica By-Products

It has been widely demonstrated that postharvest abiotic factors like ultraviolet radiation (UV), wounding/cutting, phytohormones, and altered gas composition can be applied to increase bioactive compound biosynthesis in fresh F and V [28]. However, there is less scientific evidence about the effect of such abiotic stresses on the bioactive compounds of their by-products. We have focused on two abiotic stresses as potential tools to increase the bioactive compound content of Brassicas prior to extraction since, in our opinion, they seem to be the most efficient.

### 4.1. Ultraviolet Radiation

The effect of UV on Brassica bioactive compounds (fresh-cut, sprouts, and florets) has been extensively studied [29,30,31,32,33,34,35]. But there is not much scientific evidence studying the effect of UV on Brassica by-product bioactive compounds [36,37]. Single or combined postharvest UV-B and UV-C treatments were previously proposed as an innovative and eco-friendly tool to revalorize broccoli leaves and stalk by-products through the enhancement of their main phytochemicals [36]. Particularly, a 15 kJ m^−2^ UV-B treatment induced glucobrassicin increases of 135% and 83% in leaves and stalks after 72 h at 15 °C, respectively. Additionally, broccoli leaves showed TAC increments of 120% after UV treatments, while broccoli stalks showed TPC increases of 170–420%, likely due to a higher extraction of some individual antioxidant compounds [36]. Other authors studied the effect of UV (250–400 nm, 59 and 99 kJ∙m^−2^) on the leaf waste fraction from industrial trimming of cabbage, reporting an increase of TAC, flavanols, hydroxy-cinnamates, and anthocyanins, while no changes in GLS or isothiocyanates were observed [37]. When UV radiation was combined with photosynthetic active radiation (400–700 nm, 497 kJ∙m^−2^) an increase in TAC (30%), the content of five phenolics (from 1.4 to 10-fold higher), and hydroxycinnamic acids were observed [37].

### 4.2. Wounding/Cutting

Cutting or shredding horticultural commodities affects plant metabolism by increasing secondary metabolites with antioxidant potential to fight against the abiotic stress it induces [38,39], for which reason the same trend would happen in their by-products. Recently, the effect of cutting style on the biosynthesis of phenolics and cellular antioxidant capacity in wounded broccoli has been reported [39]. In this study, the TPC increased by 45.5, 58.9, 71.2, and 98.5% in intact heads, intact florets, half florets, and shredded florets, respectively. The authors concluded that wounding stress may be a convenient way to obtain, commercially or at home, more health-promoting antioxidant compounds [39]. Other authors indicated that wounding broccoli (florets cut into four pieces), applied alone or in combination with exogenous phytohormones, can be used as an effective emerging technology to allow the accumulation of specific GLS and phenolic compounds [40]. Wounding stress was successfully applied to design a phenolic-rich carrot juice by cutting unpeeled carrots into slices and storing them for 48 h at 15 °C before blanching [41]. However, no scientific studies on the effect of cutting on Brassica by-products have been discovered, indicating that more research in this area is required in the near future.

## 5. Extraction Techniques

In the last few decades, sustainable and non-thermal techniques have been optimized to reduce costs due to conventional technologies’ high energy consumption and the degradation of thermolabile nutritional compounds and the thermal instability of several bioactive compounds during the process. Therefore, it is essential to focus on innovative non-thermal ‘Green Technologies’ such as USAE, MWAE, and EAE, among others.

Most studies are focused on fruit by-products [42], finding a lack of clear evidence related to horticultural commodities, including Brassica by-products. Due to the interest in the effect of green and non-thermal treatments on Brassica by-products for phytochemical extraction, a compilation of the scientific evidence is needed to establish the optimum treatments and conditions (extraction, addition, processing, storage, and shelf-life). Additionally, the effect of processing, including blanching, drying, homogenization, and/or grinding into powder, should be studied as pretreatments of extraction techniques.

### 5.1. Ultrasound-Assisted Extraction from Brassica By-Products

USAE consists of the propagation of ultrasonic waves in a liquid medium, inducing a longitudinal displacement of particles that create cavities in the liquid, which is called acoustic cavitation [42]. This can occur with less solvent consumption, energy, and extraction time, making it an environmentally friendly and economical technique [43].

Table 1 shows the main conditions used for USAE of bioactive compounds from Brassica by-products. According to the articles found, broccoli is the main Brassica studied, followed by cabbage, radish, cauliflower, and kale. The revalorization of Brassica by-products is mainly concentrated on leaves and stems, although there are articles focused on seeds. The frequency of USAE equipment ranged from 20 to 50 kHz. Power units depended on the equipment used, reporting values from 100 to 500 W, 50 W/L, or 0.228 W/cm^2^. The best results were achieved with an aqueous solvent. Water was used as the extractant in ten of the studies found, and in seven of them it was combined with an organic solvent (ethanol, methanol, and acetonitrile), with ethanol being the main one [44,45,46,47]. In fact, Liu et al. [48] reported a better SFN extraction with a ratio of 1:10 for water compared to 1:50 for ethyl acetate. The solid:liquid ratio in most of the studies ranged between 1:2 and 1:50, and just one of the studies found that it worked with a more diluted extract (0.06:30) [49]. The extraction temperature used was determined by the target compound or the function to be achieved by the extraction. An extraction temperature below 30 °C was best for the GLS and SFN extractions [23,46,47,48,50,51]. However, MWAE pretreatment for a short time favored SFN extraction due to the inactivation of the myrosinase enzyme and GLS-SFN conversion. Temperatures above 45 °C were used for the extraction of phenolic compounds [43,47], and in the case of protein extraction, USAE was carried out in some studies [45,52,53].

### 5.2. Microwave-Assisted Extraction from Brassica By-Products

The application of MWAE to enhance extraction consists of the ability to extract bioactive compounds from structural changes in cells due to the electric and magnetic fields generated by this technology. The conditions reported in previous studies to be considered in MWAE are summarized in Table 2. The main studied by-products came from broccoli, cabbage, and radish. Although the cv. is an important parameter to know since the phytochemical content may vary, it was not detailed in the reported manuscripts. The power intensity ranged from 130 to 400 W under atmospheric conditions, except in one study in which vacuum was applied together with MWAE to improve the extractability [18]. The solvents used for MWAE were different in each study, including water, water + ethanol, dichloromethane, nitric acid, or methanol. The most concentrated solid:liquid ratio used was 1:4 [57], and the most diluted was 0.5:31.5 [58]. Both obtained good results, because the extraction conditions (time, solvents, and temperature) were different. The temperature ranged from 20 to 90 °C, always below 100 °C to avoid bioactive compound degradation. The extraction time varied from 1 to 25 min, obtaining the best results with times of less than 20 min.

### 5.3. Enzymatic-Assisted Extraction from Brassica By-Products

EAE is based on the use of enzymes to break down the cell walls of plant material and improve the extraction yield of its bioactive compounds. The main conditions to be considered are shown in Table 3. Most of the Brassica by-products used in the studies come from broccoli, radish, cauliflower, and cabbage. Before EAE, by-products are usually pretreated by grounding and drying (oven at 45–60 °C or using a freeze-dryer), although particle size is rarely detailed. The enzymes used were determined by the compound to be extracted. The main enzymes found were cellulase, hemicellulase, protease, pectinase, and glucanase, among others. Papaioannou and Liakopoulou-Kyrikides [59] used a fungus to facilitate the β-carotene production from Brassica by-products. Other green technologies combined with EAE, such as MWAE [58] and USAE [60], have been used to increase the extraction yield prior to enzymatic rupture of the cell walls. Only half of the articles summarized in Table 3 detail the enzyme inactivation conditions; two of them used heating for a few minutes and one used refrigeration. The solid:liquid ratio ranged from 10:40 to 5:500, like other extraction methods using green technologies. Extraction time was highly variable, ranging from 8.4 to 1200 min, but the temperature was limited between 26 [59] and 68 °C [58].

### 5.4. Other Extraction Methods from Brassica By-Products

Although the most commonly cited green technologies in the bibliography have already been described, a considerable number of works have studied other technologies to extract bioactive compounds from Brassica sources. Previous research has shown that extracting pectin from broccoli stalks with 0.1 M nitric acid under reflux for 30 minutes [65] is effective, and that by-products of broccoli florets are an excellent source of glucoraphanin and phenolics after extraction in a thermostatic bath mixed with ethanol (0, 40, and 80%) for 10, 40, or 70 minutes [66]. Nevertheless, despite the recent publication of these works, only the scientific studies that include novel and green technologies to enhance the extraction ability of Brassica by-products are shown in Table 4.

As shown, four works used supercritical fluids, one used ultrafiltration, and another used pressurized liquids. All these techniques showed higher yields for recovering bioactive compounds from Brassica by-products. Nevertheless, such techniques are even more expensive than those previously described and take longer to extract the phytochemicals, although they use lower temperatures (35–60 °C) to avoid their degradation and do not require high amounts of solvents to complete the extraction. The solid:liquid ratio is not a relevant parameter in supercritical fluid technology. However, the solvent flow rate is detailed in almost all the works found as being 2 L/min. Superficial fluid technology facilitated the extraction of bioactive compounds and antioxidants, except in the work of Marinelli et al. [53], where this technology showed the worst results compared to pressurized liquid technology.

## 6. Brassica By-Products Fortification in Food Matrices

Once the main bioactive compounds have been extracted from Brassica by-products, several possibilities to fortify different food matrices have been reported or could be possible.

### 6.1. Brassica By-Products Processing Pretreatments

Blanching, drying (i.e., convective or freeze drying), and/or grinding into powder are typically used as pretreatments of extraction techniques to increase yield and stability [70]. Apart from the inhibition the enzymatic activity and retaining color and nutrients, several authors indicated that blanching could be a good strategy to enhance the recovery of phenolics and other bioactive compounds during the extraction. The phytochemical content depends on the blanching pretreatment and dehydration process applied, although dehydrated broccoli by-products are a source of pigments, including terpenoids, sulfur compounds, and phenolic compounds [71]. Recommended blanching conditions were three cycles of 2 min at 800 W, with 1 min intercalated, using a domestic microwave oven [71]. Other authors indicated that slicing cauliflower leaves prior to blanching led to higher losses of TAC during either hot water or steam blanching [72]. Water blanching led to lower retention of water-soluble antioxidants as such phenolic compounds and vitamin C [72].

On the other hand, MW hydrodiffusion and gravity (MHG) is a novel technique consisting of a combination of blanching at 100 °C and drying at 100 °C [73]. Ferreira et al. [73] reported that MHG allowed obtaining a rich dry extract from broccoli by-products cv. Parthenon, preserving polysaccharides and proteins with low moisture (12%). Furthermore, Ferreira et al. [71] previously reported the effect of MHG technique on phenolics in broccoli leaves and stalks, showing an increase of 26% TPC, preservation of GLS content, and reduction of pigments (25%). Blanching has also been used to produce chemical-free nano-fibrillated cellulose from cabbage for potential use in food formulations [74]. The authors also indicated that steam blanching of the outer leaves of cabbage was used and then dried in a hot air oven (60 °C, 8 h), and nano-fibrillated cellulose was extracted by heating (130 °C, 2 h), followed by USAE (37 °C, 1 h), or high pressures (40 MPa, 5 times) [74].

After drying, F and V by-product as a powder/flour is commonly acquired by grinding until obtaining the desired particle size [42,70]. This powder could be applied as a solid ingredient for the fortification of different products, or the key bioactive compounds can be extracted from this powder to obtain liquid extracts, which can be freeze-dried or spray-dried to obtain powders. Because of differences in diffusivity, particle size is one of the most important parameters influencing the extraction and incorporation of bioactive compounds into other food matrices. Not only must the extraction method be optimized, as stated in Section 5, but so must the drying method. The technique, the time, and the temperature should be selected to avoid the degradation of the biocompounds and to have a stable material (dry by-product) for storage until the extraction. Therefore, this process is of great importance for obtaining the best-quality extracts. Depending on the drying process, the moisture content of the sample varies and influences the extraction step [70]. Apart from the information on blanching processing, there is a lack of specific details related to drying in several pieces of scientific evidence, as previously shown in Table 1, Table 2, Table 3 and Table 4. As to Brassica by-products, other authors concluded that powdered *Brassica napobrassica* leaves sieved at three particle sizes influenced the physicochemical and functional properties of the powder. The addition of this powder to a starch suspension influenced the pasting of the suspension [75]. Other authors obtained a flour (20% leaves, 35% inflorescence, and 45% stems) by freeze-drying broccoli by-products (Naxos cv.) and grinding them (no particle size was specified). They concluded that the daily intake of a high dose of broccoli by-product flour for three weeks was safe because of the high bioavailability of GLS and had no negative impacts on the mouse’s health [76].

The use of water-blanching and grinding to obtain encapsulated stalk and floret juice powders by spray-drying using maltodextrin as a carrier was reported. The optimal processing conditions were 5% maltodextrin and a drying temperature of 220 °C. Floret juice powders showed high TPC, while stalk juice powders presented high TAC [77]. Other authors studied broccoli stems and leaves powders, from blanched and freeze-dried juice and pomace fractions, as carriers to encapsulate epigallocatechin gallate aqueous solution (EGCG). They concluded that broccoli by-product puree and pomace had higher adsorption capacities for EGCG than juice at 25 °C, making them promising carriers for delivering and stabilizing EGCG through gastrointestinal digestion [67]. The comparison between conventional and supercritical fluid extraction techniques of different leaf-stem mixes (1:1, 3:1, and 1:3) from Parthenon and Naxos broccoli by-products dried at 55 °C for 24 h and ground into uniform powder was previously studied. The results indicated that supercritical fluid extracts from broccoli by-products could potentially serve as an ingredient for cosmetic purposes [69].

Fermentation can be used for by-product revalorization to promote a circular economy and improve efficiency and profitability in the food sector [70,78]. The aim of one of these studies was to revalue broccoli stalk by designing a novel fermented food product with probiotic potential enriched in glucoerucin, indolic GLS, phenolic acids, and flavonoids [78]. Dried Brassica by-products in powder can be incorporated into whey to evaluate the effect of its supplementation on β-galactosidase enzyme production. Different concentrations (5–25% *w*/*v*) of dried cauliflower waste were cut (0.5 cm), oven-dried (50 °C), and ground (1.168 mm). A 15% increase in β-galactosidase production was observed when the cauliflower waste level was increased to 20% compared with whey alone [79].

### 6.2. Brassica By-Product Fortification in Animal Feed to Increase Functionality

The use of Brassica by-products for animal feeding (small ruminants and chicken, among others) allows their transformation into high-quality meat and milk products while promoting the development of the circular economy. Depending on the animal, Brassica by-products could be incorporated into the diet through feed and/or silage to ensure nutritional quality. The effect on milk production, composition, functional properties, and technological characteristics of goat and fermented milk has already been reported [80]. The use of silage from horticultural by-products in the diet of dairy goats has been reported to reduce feeding costs. Long-term inclusion of 40% silage from broccoli by-products, among other commodities, in the balanced diets of dairy goats yields milk suitable for yogurt and cheese fermentation. Broccoli inclusion enhanced the antioxidant properties of milk and, consequently, of fermented milks [81].

Similarly, broccoli by-products have also been incorporated into the feeding of broilers to enhance the meat quality. The incorporation of broccoli extracts with 0.075 g/kg SFN into the broiler diet increased the expression of xenobiotic and antioxidant enzymes in the jejunum of chickens, which represents a novel mechanism to improve the health of farm animals [82]. Hu et al. [83] tested different concentrations (0, 4, 8, and 12%) of broccoli stems and leaves in the corn-soybean meal of broilers during their 42 days of life. The carotenoids content and TAC of the meat improved with 4% broccoli by-products, which also increased the activities of superoxide dismutase and catalase in breast muscle by 8 and 12%, respectively.

### 6.3. Brassica By-Products Fortification in Several Food Matrices

This section has been focused on the scientific evidence related to the use of Brassica by-products for fortification and incorporation into human food matrices. Table 5 includes information about the characteristics of Brassica by-products (drying technique, particle size, and cv.), extraction technique (US, maceration), formulation, incorporation method (liquid extracts, powders), and benefits tested after incorporation (shelf-life, bioactive compound fortification). Table 5 was divided into categories related to Brassica species by-products: broccoli, kale, cabbage, and cauliflower, broccoli and cauliflower being the most common. Broccoli by-product incorporation has been reported in several food matrices in powders, liquid extract, and/or encapsulated, among others: dressing [84], bakery products [85,86,87,88,89,90], dairy products [91,92], snacks [87], fish products [53], and beverages [93], with bakery products being the most common. Broccoli by-product extracts, mostly in solid form, can be considered a promising source for designing new foods with interesting techno functional and functional properties. A pesto sauce was enriched with kale by-products, obtaining several benefits (Table 5) [12]. This research concluded that more studies should be conducted with non-thermal blanching to minimize myrosinase inactivation [12]. A cabbage leaves by-products powder was added into the sponge cake flour to substitute 10% and 20% of wheat flour, enhancing some bakery properties (Table 5) [94]. Some scientific evidence related to the incorporation of cauliflower by-product focused on replacing several ingredients such as wheat flour in snacks [95], carrageenate of vegan paté [96], dried whole egg and starch in a quiche [96], and xanthan in tomato sauce [96]. The aim of the remaining evidence was the enrichment of several food matrices with cauliflower by-products: chicken soup [97], pork patties [98], and apple juice [52].

## 7. Conclusions and Future Perspectives

Green technologies used to extract the main biocompounds from Brassica by-products and their possible application to fortify new foods have been thoroughly reviewed. The extraction yield depends on the raw material (cv., moisture, part of the plant, etc.), the applied pretreatment (drying technology, particle size, abiotic elicitors such as UV or wounding, etc.), and the key compound to be extracted (fiber, phenolics, isothiocyanates, GLS, etc.). Specific conditions and parameters must be monitored during the extraction process, and their optimization must be studied. Although most of the evidence found is related to USAE and MWAE as the best extraction methods, there are studies suggesting others like, EAE, and novel technologies such as supercritical fluids, ultrafiltration, or pressurized liquids, which may involve a higher cost. Thus, in future studies, energy efficiency/consumption, environmental impact, and predictive models must be included to optimize the phytochemical extraction. As the main conclusion, through the addition of Brassica by-products and their incorporation into new fortified products, it will be possible to revalorize the Brassica losses generated during the first steps of the food production chain, developing new products with potential health benefits while reducing their environmental impact within a circular economy framework.

## Figures and Tables

**Table 1:** Ultrasound conditions (frequency, power parameters, solvent, time, and temperature) for the extraction of bioactive compounds from Brassica by-products.

By-Product Characteristics	F (kHz)	Power Parameters	Solvent	S:L Ratio (w:v)	T (min)	T (°C)	Other Information	Main Findings	Ref.
Radish seeds cv. IPR 11 Particle size information NA	25	165 W	EtOH	1:12	20–60	30–60	USAE bath with indirect contact. After the extraction, seeds were separated by filtration, and the excess solvent was removed until reaching a constant weight.	The maximum yield (25%), a greater amount of phytosterols and tocopherols, and, consequently, greater oxidative stability.	[54]
Red radish cv. information NA Freeze dried 1–2 mm pieces	NA	138–358 W	H_2_O	0.06:30	30–120	45	Before USAE by pulse cycles of 5 s on and 1 s off, extraction of anthocyanins was performed.	High-energy USAE treatment (120 min at 286–258 W) is adequate to enhance TAC but does not preserve anthocyanins.	[49]
Broccoli leaves, stems, and inflorescences cvs.: ‘TSX 007′, ‘Monaco’, ‘BRO 2047′, ‘Parthenon’, and ‘Summer Purple’ Dried (45 °C, 48 h) Particle size information NA	NA	NA	80% EtOH	10:60	60	45–50	Excess EtOH was removed by heating it at 37 °C in a rotary evaporator under vacuum. The resulting aqueous extracts were combined and lyophilized.	Extraction yield of 13.4–16.3% dw. High TAC and chlorophylls and phenolics (mainly kaempferol and quercetin glucosides) in leaf extracts (‘Summer Purple’) and high GLS content in inflorescence extract.	[24]
Broccoli leaves, stems, and inflorescences cv. Parthenon Dried (45 °C, 24–48 h) Particle size information NA	NA	220 V 360 W	H_2_O	1:50	60	NA	Before USAE, the mixture was heated for 16 min at 121 °C. After US, four times its volume of ethanol was added, and after 12 h of incubation, it was dried at 45 °C in a forced-air oven.	USAE did not manage to modify the neutral sugar profile.	[55]
Broccoli by-products cv. information NA Dried (35 °C, 48 h) Particle size information NA	25	50 W/L	H_2_O	1:10	60	15	The extract was dried at 30 °C in a vacuum oven. The residue was mixed with water and recovered by centrifugation (6000 rpm × 10 min).	USAE extracted more bioactive compounds than supercritical fluids but not as many as pressurized liquid.	[53]
Cauliflower by-products cv., drying, and particle size information NA	NA	175 W	H_2_O (pH 11)	1:4	15	NA	The crude fiber and insoluble protein were removed from the extract first with 3 layer gauze and then by centrifugation (4000 rpm × 30 min).	Extraction yield of 53.1% and 12.066 g of soluble leaf protein kg^−1^.	[23]
Cauliflower by-products Blanching cv. information NA Dried (50–55 °C overnight) Particle size 0.5 mm	24	400 W	H_2_O 70% MeOH 80% Ac	50:100	0–10	NA	Amplitude USAE from 20–100%. After US, centrifugation at 1500× *g* for 15 min, and the pellet was centrifuged with 100 mL of solvent. Both supernatants were collected, combined, and filtered under vacuum conditions.	The amplitude affected the extraction of isothyocyanates (80% amplitude for 3 min) and phenolics (100% amplitude for 3 min).	[52]
Rapeseed meal cv., drying, and particle size information NA	28	0.228 W/cm^2^	H_2_O	1:30	41.48	NA	Other extraction conditions were pH 11.71 and USAE power 40%.	High protein yield of 43.3% and nitrogen solubility of 18.1%.	[44]
Broccoli cv., drying, and particle size information NA	40	500 W	Ch 80% EtOH Ac	100:500	60	40	Extracts were combined to metal-organic framework nanocubes. They were dispersed by an ultrasonic probe in 100 mL, then triethylamine as a capping agent was added, and the mixture was agitated and heated for 12 h at 130 °C.	Broccoli extract combined with MOF-5-NCs showed synergistic activity against *P. aeruginosa* bacteria in standard and clinical strains.	[43]
Kale cv. information NA Convective dryer (39 °C) Particle size information NA	20	100 W	80% EtOH	2:40	60	60	USAE in two cycles of 30 min Extracts were filtered, combined, and evaporated. The residues were dissolved in methanol and filtered.	High isolation of phenolic acids and high yield of biocompounds in short time and reduced solvent volume with easy handling.	[45]
Broccoli seeds cv., drying, and particle size information NA	NA	200–500 W	H_2_O EA	1:10–1:50	5–40 s	25–35	Before USAE, broccoli seeds were treated in a MWAE oven for 1–4 min at low power.	The highest SFN formation was under a MWAE pretreatment of 3 min and a US treatment of 20 s, 500 W, and 1:10 for water or 1:50 ethyl acetate.	[48]
Broccoli stems and leaves cv. information NA. Dried (30–35 °C, 48 h). Particle size information NA	25	50 W/L	H_2_O	1:10	60	NA	After homogenization, the extract was dried at 30 °C in a vacuum oven. The residue was mixed with water (25 mL) and recovered by centrifuging at 6000 rpm for 10 min.	High-quality extract in terms of antimicrobial efficacy against *Pseudomonas* spp. and *Candida krusei*.	[56]
White cabbage cv. information NA Oven-dried (60 °C, 72 h) Particle size information NA	40	132 W	60% EtOH	2:10	120	30–70	Ultrasonic intensity of 0.46 W/cm^2^. The obtained extracts were hydrolyzed before analyzing.	Richer extract at 30 °C. Antimicrobial activities only of the hydrolyzed extracts.	[46]
Broccoli heads cv., drying, and particle size information NA	23	NA	H_2_O	1:20	1–12	25–60	Amplitude was set at 135 µm.	Higher myrosinase inactivation and SFN content at 60 °C for 4 min. Activation energy was 3.6-fold lower regarding traditional blanching.	[50]
*Camelina sativa* oil cv., drying, and particle size information NA	35	60–120 W	40–80 EtOH	1:5–1:15	10–20	30	USAE in 2–4 cycles of 5 min each. A solid-phase extraction procedure to obtain an extract rich in GLS and to perform cellular assays.	High-GLS extraction with 65% EtOH, 1:15, and 10 min. The purified extract (800 mg from 10 g) showed chemopreventive action against colorectal cancer cells.	[47]
Thirty-six *Brassica oleracea* var. *acephala* accessions Dried in an oven (105 °C) or freeze-dried Particle size information NA.	40	300 W	80% MetOH	0.03:1.5	30	20	After USAE, extracts were centrifuged at 15,000× *g* for 5 min.	Higher GLS content, TAC, TPC, and sugars with freeze-dried samples and USAE compared with hot extraction.	[51]
Cabbage leaves, fresh and steamed (100 °C, 2 min) cv., and drying info NA Particle size 1.7–2.55 mm.	37	320 W	H_2_O	5:50	40	NA	Absorbed US power of 0.03 W/g extraction + MWAE or vaccum.	Higher glucoraphanin content with USAE + vacuum or MWAE More effective (87%) when leaves were steamed, presenting higher myrosinase inactivation.	[18]

NA: Data not available; cv.: cultivar; Ac: acetone; EA: ethyl acetate; Ch: chloroform; TPC: total phenolic content; TFC: total flavonoid content; TAC: total antioxidant capacity; GLS. Glucosinolates; SFN: sulforaphane; S:L: solid:liquid.

**Table 2:** Microwave conditions (power parameters, solvent, time, and temperature) for the extraction of bioactive compounds from Brassica by-products.

By-Product Characteristics	Power (W)	P	Solvent	S:L Ratio (w:v)	T (min)	T (°C)	Other Information	Main Findings	Ref.
Purple-heart radish cv. information NA. Dried in the oven (60 °C) Particle size 117-μ*m*.	NA	Atm	H_2_O andEtOH	0.5:31.5	20	70	Twenty grams of broccoli powder were pre-extracted with petroleum ether II at 80 °C for 6 h.	Polysaccharide yield (29%) was higher than hot (~24%) and USAE (27%) extraction.	[58]
White cabbage leaves are chopped. cv. information NA. Fresh or dried with a hot air dryer (60 °C) Particle size information NA.	130–390	Atm	DCh H_2_O	5:50	1–5	22–38(DCh)22–98(H_2_O)	After extraction with a domestic MW oven, the extract was filtered and dehydrated using the rotary evaporator at 30 °C (for DCh) or 45 °C (for H_2_O).	Higher SFN yield in less time. Higher MW powers resulted in a shorter extraction time.No differences between fresh and semi-dried samples, nor between the solvents used.	[61]
Broccoli florets, stems, and leaves. cv., drying, and particle size information NA.	NA	Atm	40–80% MetOH	1:20	10–20	55–75	After extraction, the mixture was centrifuged for 20 min at 10,350 rpm and 4 °C. The supernatant was filtered and stored at −20 °C.	The optimum conditions were 74.5, 80, 80% MetOH, 15.9, 10, 18.9 min, and 74.5, 73.3, 75 °C for stalks, leaves, and florets, respectively. Increased the phenolic yield up to 65.3, 45.70, 133.6% for stalks, leaves, and florets, respectively, in less time.	[62]
Purple and white cabbages cv. information NA. Sun-dried. Particle size 80–100 µm.	200–400	Atm	NAc	1:4–1:7	10–25	60–90	After extraction, the extract was completed with 10 mL.	Optimum conditions: 201 W at 60 °C for 10 min at a 1:4 ratio. A polynomial regression was the best-fitting model.	[57]
Cabbage leaves (1.7–2.55 mm) cv. information NA. Fresh and steamed. (100 °C for 2 min). Particle size information NA.	180	Atm 70 kPa	H_2_O	5:50	10	NA	Combined with USAE	Higher glucoraphanin content using vacuum MWAE with USAE than atmospheric MWAE. More effective (87%) when leaves were previously steamed, and a higher inactivation of the myrosinase enzyme.	[18]

NA: Data not available; cv.: cultivar; SFN: sulforaphane; NAc: Nitric acid; DCh: Dichloromethane; Atm: Atmospheric; P: pressure; S:L: solid:liquid.

**Table 3:** Enzymatic conditions (enzyme, pressure, time, and temperature) for the extraction of bioactive compounds from Brassica by-products.

By-Product Characteristics	Combined with	Enzymes	Inactivation Enzymes	S:L Ratio (w:v)	T (min)	T (°C)	Main Findings	Ref.
Purple-heart radish cv. information NA. Oven-dryer (60 °C).	MW	Papain	NA	1:55–1:65	8.4	68	EAE combined with MWAE facilitated cell rupture and enzymolysis, improving the extraction yields and shortening the extraction time.	[58]
Broccoli by-products (leaves, stems, and inflorescences). cv. Parthenon Forced-air oven dryer (45 °C, 24–48 h)	NA	Cellulase	Cooled at room temperature	1:50.8	120	50	Decreased the sugar content and increased the uronic acid content. Non-extractable phenolics were found higher in inflorescences and increased with EAE and TAC.	[55]
Radish root ground with a mortar. cv. and drying information NA.	US	Cellulases Pectinases Amylases Glucanases Hemicellulases	Few minutes at 90 °C	10:40	66–84	46–64	Higher TAC with the highest extraction of TPC.	[60]
Canola (*Brassica napus*) oil pressing residues. Particle size: 0.5 mm cv. and drying information NA.	NA	Protamex^®^ Alcalase^®^ Viscozyme^®^ Phyzyme^®^	NA	1:10	240–1200	45–50	The applied enzymes effectively enhanced the solubility of proteins, despite the lower yield of crude proteins compared to the alkaline extraction (40–82 vs. 91 g/100 g dw).	[63]
Cauliflower florets and leaves cv. information NA Pre-extraction with 96% ethanol (1:5) for 30 min at 100 °C. Residue was dried at 40 °C.	NA	Proteases Cellulases Endopolygalacturonase II Rhamnogalacturonan hydrolase Pectin methyl esterases Rapidase Liq+	10 min at 100 °C	5:500	240	50	Higher methoxy pectins of high molar mass were extracted with three enzyme mixtures. Health benefit pectic oligosaccharides were obtained after pectin extraction. Seventy percent of the by-products were consumed to extract two products of interest.	[64]
Cabbage (91.5% humidity)	NA	*Blakeslea trispora* (mould)	NA	1:10	NA	26	Higher biomass accumulation and carotenoid production.	[59]

NA: Data not available; cv.: cultivar; TPC: total phenolic content; TAC: total antioxidant capacity; S:L: solid:liquid.

**Table 4:** Other green technologies used for the extraction of bioactive compounds from Brassica by-products.

By-Product Characteristics	Green Technology Used	S:L Ratio (w:v)	T (min)	T (°C)	Other Parameters to Be Monitored	Main Findings	Ref.
Broccoli leaves, stems, and inflorescences. cv. ParthenonDried in a forced-air oven (45 °C, 24–48 h).	Supercritical fluids using CO_2_	NA	120	45–55	Dynamic extraction. Flow: 2 L/min. Three hundred bar at 55 °C or one-hundred and fifty bar at 45 °C.	The content of non-extractable phenolics and TAC increased and were higher in inflorescences.	[55]
Broccoli by-products. Dried (35 °C, 48 h).	Supercritical fluids using CO_2_	NA	140	35	Two pumps: (i) Supercritical CO_2_ (ii) Organic co-solvent (20% EtOH). 150 bar Flow: 2 L/min	Presented the worst results regarding the extraction of bioactive compounds.	[67]
Broccoli by-products. Dried (35 °C, 48 h)	Pressurized liquid	15:25	10	60	Steps: (i) Filling the cell with 70% EtOH, 2–3 min; (ii) Upto 1500 psi; (iii) Five minutes at 60 °C + 5 min extraction; (iv) Static and 30 s depressurization; (v) Washing the cell for 50 s; (vi) Purge the solvent with N_2_ 2 min. Drying in a vacuum oven (30 °C).	The highest content of bioactive compounds and TAC.	[53]
Yellow mustard flour (30.7% oil, 30.9% protein, 4% ash, and 9% fiber).	Ultrafiltration	NA	NA	25	Before ultrafiltration, defatting was carried out with hexane. Film composite membrane (150–300 Da, pH tolerance range 2–10 at 25 °C, max. Tª of 80 °C, and pressure of 40 bar).	In acidic conditions, 77% of the phenolic compounds were recovered. Combination of diafiltration with nanofiltration was beneficial only when processing under acidic conditions.	[68]
Broccoli stems and leaves Dried (30–35 ℃, 48 h).	Supercritical fluids using CO_2_	NA	140	35	Two pumps: (i) Deliver solvent; (ii) Organic co-solvent (100% EtOH). 50 bar Flow: 2 L/min Drying in a vacuum oven (30 °C)	High-quality extract in terms of antimicrobial efficiency against *Pseudomonas* spp. and *Candida krusei*.	[56]
Broccoli stems and leaves cv. Parthenon and Naxos.	Supercritical fluids using CO_2_	NA	NA	NA	Two pumps: (i) Supercritical CO_2_; (ii) Co-solvent (20% EtOH).	High yield of β-carotene, phenolic compounds, chlorophylls, and phytosterols. Great TAC. Reduced organic solvent consumption.	[69]

NA: Data not available; cv: cultivar; TAC: total antioxidant capacity; S:L: solid:liquid.

**Table 5:** Application of Brassica species (by-product characteristics and incorporation method/formulation) and their benefits in different food matrices.

*Brassica* Species	Matrix	By-Product Characteristics	Formulation Incorporation	Benefits	Ref.
Broccoli (*Brassica oleracea* var. *italica*)	Salad dressing recipes	Stems and leaves cv. Marathon No pre-blanching Freeze-dried Grounded fine powder	Powder:lemon juice:oil (olive, hazelnut, or sunflower) ratio (1:2.5:7.5; w:v:v).	Higher bioaccessibility of polyphenols from broccoli in an oil matrix.	[84]
	Durum Pasta	Leaves cv. Sebastian Blanching Freeze-drying Particle size ≤0.60 mm	Durum semolina flour, water, olive oil, and salt. Leaves powder: 0–5%.	Decreased cooking time and water absorption. Increased the swelling index. Firmness and total shearing force decreased. Greener than control. Higher dimethyl sulphide and mineral content. No effect on overall acceptance.	[86]
	Gluten-Free Sponge Cakes	Mature leaves cv. Sebastian Blanching in hot water Freeze-dried Particle size ≤0.60 mm	Potato and corn starch, eggs, sugar, oil, salt, and baking powder. Leaves powder: 0–7%.	Good source of free amino acids. Promising product for a gluten-free diet.	[85]
	Powders and extruded snacks	Broccoli pomace Steam blanching Freeze-drying Particle size: 800 µm sieve	Dried and wet pomace are used for extrusion. Vegetable powder:rice flour ratio (100:0, 80:20, 60:40, 40:60, 20:80, and 0:100). Maximum wet pomace: 3%.	Enhancement of the nutritional properties. Powders were richer in fiber but contained less total carbohydrates. A reduced expansion of extruded snacks with increasing vegetable levels in the formulation.	[87]
	Gluten-free bread	Mature leaves cv. Sebastian Blanching in hot water Freeze-dried Particle size ≤0.60 mm	Corn starch, potato starch, sugar, fresh yeast, pectin, rapeseed oil, salt, and water. By-products (5%) instead of corn starch.	Higher content of proteins and minerals. Improved specific volume and bake loss. Improved TAC and anti-aging activity.	[88]
	Gluten-free mini sponge cake	Mature leaves cv. Sebastian Blanching in hot water Freeze-dried Particle size ≤0.60 mm	Consists of 30.6% potato and 7.8% corn starches, 43% egg, 14% sugar, 3.7% sunflower oil, 0.2% salt, and 0.7% gluten-free baking powder. The inclusion was: 2.5–7.5% (*w*/*w*)	Increase of firmness. No changes in sensorial quality. Sample with 2.5% was distinguished.	[89]
	Gluten-free mini sponge cake	Mature leaves cv. Sebastian Blanching in hot water Freeze-dried Particle size ≤0.60 mm	Consists of 30.6% potato and 7.8% corn starches, 43% egg, 14% sugar, 3.7% sunflower oil, 0.2% salt, and 0.7% gluten-free baking powder. The inclusion was: 2.5–7.5% (*w*/*w*).	Increased GLS content and TAC. Optimal improvement with addition of 2.5% as starch substitute.	[90]
	Deep fat-fried fortifiedtortilla chips	Waste cv. Plenck Dehydrated wastes Particle size <250 µm	Broccoli flour: 2–8%.	Increased contents of protein (from 8.1 to 9.5%), crude fiber (from 1.9 to 3.1%), lysine (from 25.6 to 35.1 g kg^−1^), and calcium (from 0.45 to 0.73 g kg^−1^). A 10.5% lower final oil content.	[99]
	Primosale cheese	Dried (30 °C, 48 h) Fine powder	50 and 100 g kg^−1^	Better nutritional properties, friability, and adhesiveness.	[91]
	Spreadable cheese	Stalks and leaves Dried (30 °C, 48 h) Fine powder	50 and 100 g kg^−1^	Increased TPC, TFC, and TAC.	[92]
	Fish burgers	Dried (35 °C, 48 h) Hammer mill	Extracted by USAE. Spray-dried: maltodextrins, wall material (10–30%), the core/wall material ratio (1:2, 1:5, 1:10, 1:20), and T: 80–170 °C. Minced fish is mixed with 5% *w*/*w* of microencapsulated extract.	Increased TPC and TAC, even if cooked.	[53]
	Beer	Powder	Supplementation of 0.1% powder (*w*/*v*). After 3 days at 10 °C, the by-product was removed and the beers remained in fermentation until day 14, which then ended.	Higher SFN content (2.54 mg/L, prior to bottling). SFN remained stable until bottling, when concentrations decreased by >50%. After 150 days, the SFN content was 0.30 mg/L in beers supplemented with powder.	[93]
Kale (*Brassica oleracea* var. *sabellica*) + Broccoli (*Brassica oleracea* var. *italica*)	Kale pesto sauce	Leaves Vaccum-packaged Blanching in a water bath	Kale pesto with kale leaves. Kale pesto with kale and bimi broccoli by-products. Mustard was included.	No influence on sensory quality. Glucoraphanin content was enhanced. Including mustard showed better microbial quality and color preservation after 20 days at 5 °C, without sensory alterations.	[12]
Cabbage (*Brassica oleracea* var. *capitata*)	Gold nanoparticles	Stems cv. Galega Shade-drying at room temperature	Aqueous extraction (1:2 w:v), (100 °C, 15 min) + frozen. Different volumes of an aqueous solution of HAuCl4 (0.01 M) were added to a fixed volume of extract.	Higher TPC and TAC.	[100]
	Sponge cake	Leaves White cabbage Blanching in hot water: Cabbage:water 1 g per 7 cm^3^ Dried in the oven (80 °C, 6 h) Particle size <200 µm	Eggs, sugar, and wheat flour.A double mixing procedure: dividing the whipping of egg whites and egg yolks. A by-product was added to substitute between 10 and 20% of the wheat flour.	Lower springiness of cakes and crumb tenderness. The structure was stable at high loads (lower shrinkage). Nutritional value decreased.	[94]
Cauliflower (*Brassica oleracea* var. *botrytis*)	Ready-to-eat snack	Florets, curd, stem, and leaves Oven-dried (80 °C, 10 h) Particle size: 0.5 mm mesh	Consists of 35.6% wheat flour, 20% corn starch, 10% oat flour, 10% egg whites, 10% milk powder, 3% onion powder, 5% tomato powder, 5% carrot powder, 0.1% dill, 0.1% mint, and 0.4% salt. Wheat flour was replaced with dried cauliflower: 5–20%.	Levels of 5–20% increased dietary fiber, protein content, and water absorption index. Significant effects on the expansion indices, bulk density, color, and total cell area. The taste panel acceptability score showed that cauliflower by-products could be added up to 10%.	[95]
	Commercial chicken soup	Leaves and stems	Extraction by boiling water (1:4 *w*/*v*) (1 h) and freeze-dried. Addition: 2.5–10 mg extract/mL soup.	The best concentration was 5 mg of extract/mL of soup. TAC increased between 3.5- and 13-fold (ABTS·+ assay) as well as between 23- and 85-fold (FRAP assay).	[97]
	Carrot paté	Floret/curd and stem Convection oven (between 40 and 75 °C) Particle size <100 µm	Consists of 62.5% pulverized carrot, 15% whole egg, 3.4% margarine, 9% water, 1.2% lemon juice, 1.7% sugar, 3% milk powder, 2.7% starch, 0.8% carrageenan, 1.4% salt, 0.03% riboside, and 0.08% pepper. Carrot paté (1.8%) and carob-carrageenan (0.2%) were replaced by 2% (*w*/*w*) by-products.	Products underwent discoloration (more yellowish) and a decrease in firmness and adherence, which could limit their potential as fiber supplements. Hardness and adherence decreased in floret and stem formulations.	[96]
	Quiche ‘Lorraine’	Floret/curd and stem Convection oven (between 40 and 75 °C) Particle size <100 µm	Consists of 29.3% water, 22% whole eggs, 20% cream, 8% ham, 8% onions, 6% cheese, 3% milk powder, 2% starch, 1% oil, 0.6% salt, and 0.1% pepper. Consists of 2% cauliflower fiber instead of dried whole egg (1.5%) and starch (0.7%).	The quiche containing florets and stems had a cauliflower flavor, although the overall texture was less gelled, especially for the stem samples, but the color was not affected. The quiche was considered suitable for addition of fibers.	[96]
	Meat products	Floret/curd and stem Convection oven (between 40 and 75 °C) Particle size <100 µm	Beefburgers were prepared by adding 2% (*w*/*w*) of a fiber preparation.	Improvement of the yield (10%) for stalk and floret samples. Firmness was improved when stem and floret were added.	[96]
	Bechamel sauce	Floret/curd and stem Convection oven (between 40 and 75 °C) Particle size <100 µm	Consists of 71.1% water, 8% milk powder, 4% margarine, 3.1% flour, 1.5% starch, 1% egg yolk, 10.3% fat, 0.77% salt, and 0.09% pepper. Inclusion: 3% (before or after cooking).	Viscosity increased when cauliflower fiber was added before cooking (in the case of the floret and mainly the stem). The effectiveness of supplementation depends on the time of their incorporation (before or after cooking). Modifications to color, texture, and cauliflower flavor in sensorial analysis.	[96]
	Tomato sauce	Floret/curd and stem Convection oven (between 40 and 75 °C) Particle size <100 µm	Consists of 69.4% water, 18% tomato concentrate, 6% carrot puree, 1% onion powder, 1% flour, 0.5% starch, 0.07% garlic powder, 0.75% salt, 0.05% pepper, 1% sugar, 2% olive oil, and 0.25% xanthan. Inclusion: 2% (*w*/*w*) of fiber-enriched materials and 0.15% xanthan.	It was designed to test whether cauliflower could partially substitute for xanthan as a thickening agent. The samples presented a granular texture, which limited their use except for their incorporation in ‘bolognese’ type sauces.	[96]
	Pork patties	Leaves Dried in a vacuum oven (45 °C, 8 h)	Consists of 50 g of califlower leaves ground + 500 mL (80% EtOH). Incorporation: 2.5–10 g/kg by-product extracts, or 0.2 g/kg BHA.	Higher TPC and DPPH values and lower TBARS values and protein carbonyl contents. Microbial growth was retarded.	[98]
	Apple juice (total sugar of 9.2 g/100 mL)	Steam and leaves Blanching with hot water Dried (10 min, 50–55 °C) Particle size: 0.5 mm mesh	Extracted by USAE (Table 1). Cauliflower extracts: 0–40%.	They are appropriate, containing up to 10% extract. Nutritional value was improved by enhancing isothiocyanates. Differences in smell and taste with 20% and 40% extracts.	[52]

NA: Data not available; cv.: cultivar; Ac: acetone; EA: ethyl acetate; Ch: chloroform; TPC: total phenolic content; TFC: total flavonoid content; TAC: total antioxidant capacity; GLS. Glucosinolates; SFN: sulforaphane; T: temperature.

## Data Availability

Not applicable.
